# Efficacy of using the mechanochemical endovenous ablation to treat chronic venous ulcers: case report and literature review

**DOI:** 10.1590/1677-5449.202301732

**Published:** 2025-11-10

**Authors:** Arthur Souza Magnani, Thiago Faiad Name Villari, Alexandre de Azevedo Melo, Marcela Juliano da Silva, Dafne Braga Diamante Leiderman

**Affiliations:** 1 Hospital Israelita Albert Einstein HIAE, São Paulo, SP, Brasil.; 2 Hospital Estadual Ipiranga, São Paulo, SP, Brasil.; 3 Universidade de São Paulo USP, Faculdade de Medicina, São Paulo, SP, Brasil.; 4 Instituto de Assistência Médica ao Servidor Público do Estado de São Paulo IAMSPE, São Paulo, SP, Brasil.

**Keywords:** venous ulcer, mechanochemical ablation, flebogrif®, polidocanol foam

## Abstract

This case report presents the successful treatment of a 47-year-old male patient with a venous ulcer of the leg associated to deep and superficial venous insufficiency. The patient presented with a non-healing venous ulcer on his leg, ocher dermatitis and heaviness of the leg, which interfered with work activities. Duplex ultrasound revealed deep venous insufficiency (popliteal vein), incompetence of the great saphenous and indirect perforating veins. The treatment involved ablation of the great saphenous vein using Flebogrif®, a specific mechanochemical ablation device. Following the treatment, the patient experienced significant improvement in his symptoms and venous ulcer was completely healed 9 weeks after the procedure and one year after that. Duplex ultrasound performed after the treatment confirmed the successful closure of the great saphenous vein. This case report highlights the effectiveness of mechanochenical ablation in the management of venous ulcers, even when deep venous insufficiency is involved.

## INTRODUCTION

The estimated prevalence of chronic venous disease varies according to the geographic region; it is higher in Western countries, where it can affect up to 40% of the female adult population and 17% of adult males.^1^ In Brazil, around 62% of women and 37% of men over the age of 30 years have the disease.^2^ Established risk factors include female sex, age, pregnancy, family history of chronic venous disease, obesity and occupations that demand prolonged standing.^3^

New techniques to treat great saphenous vein incompetence have been developed with the objective of improving the postoperative recovery observed after conventional vein stripping. Radiofrequency or laser thermal ablation is currently considered the gold standard, with studies demonstrating technical success rates ranging from 90 to 100% of cases,^4^ corroborating its indication in the Brazilian Society of Angiology and Vascular Surgery’s Chronic Venous Disease Guidelines.^5^ Polidocanol foam sclerotherapy provokes chemical injury to the vessel wall, but has a higher recanalization rate in saphenous veins with diameters exceeding 6 mm (from 51.2% to 74.2% within 3 years).^4^

Mechanochemical ablation (MOCA) was developed with the objectives of achieving a painless treatment, since it does not employ heat or tumescence, and improving on the technical success rate observed for treatment with sclerotherapy in isolation. MOCA combines the chemical injury caused by the foam with mechanical injury caused by traction with retractable radial hooks, in the case of the Flebogrif® device (Balton, Warsaw, Poland), or by rotating wires, in case of the Clarivein® device (Merit Medical Systems Inc., South Jordan, United States).^4^

This article reports the case of a patient with a lower limb venous ulcer who was successfully treated using the Flebogrif®. This case report was assessed and approved by the relevant Ethics Commission (CAAE 80173524.0.0000,0071, consolidated opinion number 6.859.125) and adheres to the CAse REport (CARE) guidelines.

## CASE REPORT

The patient was a 47-year-old, Black male smoker with hypertension, taking amlodipine and losartan, who had had an ulcer on the right leg for 2 months and had no personal history of deep venous thrombosis (DVT). The ulcer had developed spontaneously, expanding progressively, and was accompanied by edema, hyperchromia of the limb, and intense pain, causing insomnia and compromising his occupational activities, which required him to remain standing for long periods.

He was treated clinically with antibiotic therapy in cycles, with cefazolin, amoxycillin-clavulanate, and clindamycin in conjunction with ciprofloxacin, in addition to topical creams such as Diprogenta® (Anápolis, Brazil) and collagenase, as instructed by general practitioners, but with no improvement in his condition.

**Figure 1:**
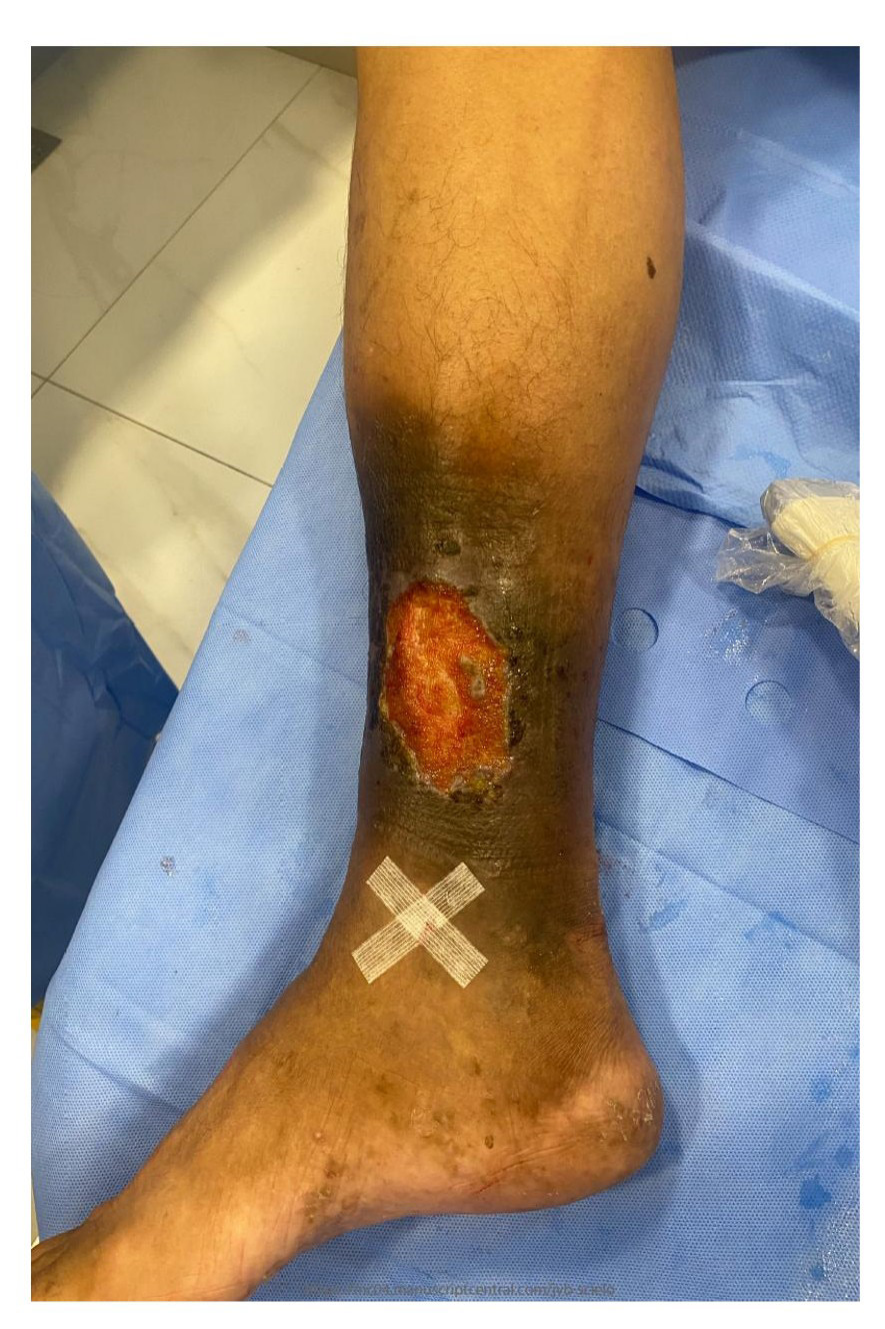
Venous ulcer on the medial aspect of the ankle. The steri-strip indicates the great saphenous vein puncture site chosen for the treatment with the Flebogrif®.

On physical examination, varicose veins were observed on the distal third of the right leg, with ochrodermatitis, edema, and an ulcer on the medial aspect of the ankle, with a maximum diameter of approximately 10 cm. The ulcer did not appear to be ischemic or infectious, with good granulation tissue (Figure 1). All pulses (femoral, popliteal, and tibial) were palpable bilaterally.

**Figure 2:**
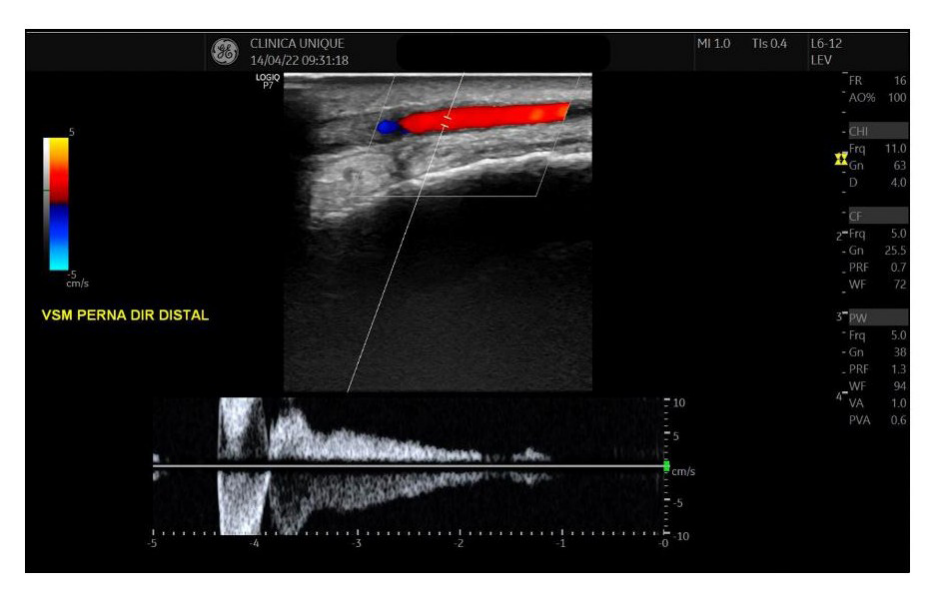
Duplex ultrasound image from the initial consultation demonstrating significant reflux in the great saphenous vein. GSV: Great saphenous vein

Venous duplex ultrasound of the lower limbs was performed in a standing position, revealing incompetence of the superficial and deep systems (reflux exceeding 1 second in the popliteal vein), with insufficiency at the saphenofemoral junction and along the path of the great saphenous vein during Valsalva maneuvers and distal compression (Figure 2). The diameters of the great saphenous vein in the proximal and distal thigh were 0.80 and 0.67 cm respectively. The diameter of the great saphenous vein in the leg was 0.50 cm, both in its most proximal segment and in the ankle. Incompetent tributary and perforator veins were also observed within the same territory as the ulcer.

**Figure 3:**
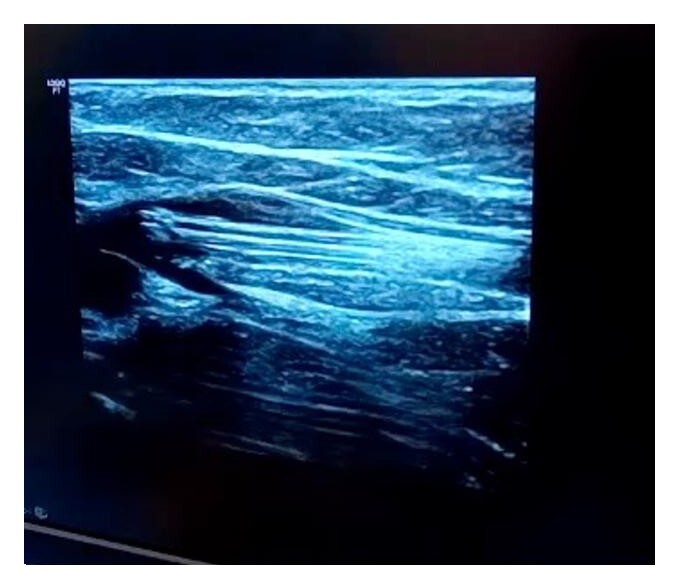
Intraoperative ultrasound image showing the Flebogrif® catheter with hooks open at the level of the saphenofemoral junction.

Initially, analgesia was administered with dipyrone, naproxen, and pregabalin, combined with a dressing including an oil-based lotion for 3 days, plus micronized diosmin + hesperidin (Daflon®, Laboratórios Servier do Brasil, Rio de Janeiro, Brasil) at a dosage of 1,000 mg per day. The decision was taken to treat the right great saphenous vein with Flebogrif® in an outpatients setting. An anesthetic bleb with lidocaine 2% and no vasoconstrictor was administered exclusively at the site chosen for ultrasound-guided puncture of the right great saphenous vein, at the level of the medial malleolus. Next, the site that had been anesthetized was punctured again, with the aid of ultrasound and a guidewire, and the Flebogrif® catheter was advanced up to the saphenofemoral junction, where the retractable hooks were released (Figure 3).

The standardized Tessari technique for 3% polidocanol foam was used (1 mL of polidocanol 3% mixed with 4 mL of air). The foam was injected as the Flebogrif® catheter was withdrawn. Around 1 mL of foam was administered for each 5 cm vein length, totaling 13 mL of foam, with no intercurrent conditions. The varicosed tributary at the level of the ulcer was punctured directly with the aid of ultrasound and injected with 2 mL of polidocanol 1% foam, using the Tessari technique (1 mL of polidocanol 1% mixed with 4 mL of air). The total procedure duration was 7 minutes and the final control ultrasound showed that the foam was distributed uniformly along the entire length of the great saphenous vein and also along the local tributaries and perforators.

At the end of the procedure, dressings were applied using HydroClean® (Hartmann Brazil, São Gonçalo, Brazil) and transparent film plus a rigid high-compression, thigh-length, elasticated stocking (30-40 mmHg). After 3 days, this dressing was swapped for Vaseline-impregnated gauze mesh, maintaining daily use of the elasticated stocking.

**Figure 4:**
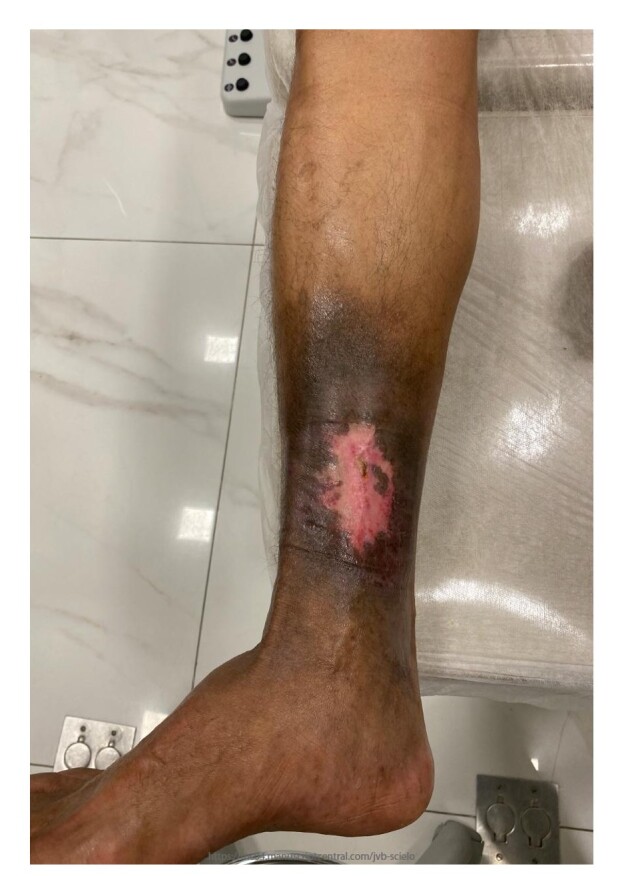
Healed ulcer 9 weeks after the mechanochemical ablation procedure.

**Figure 5:**
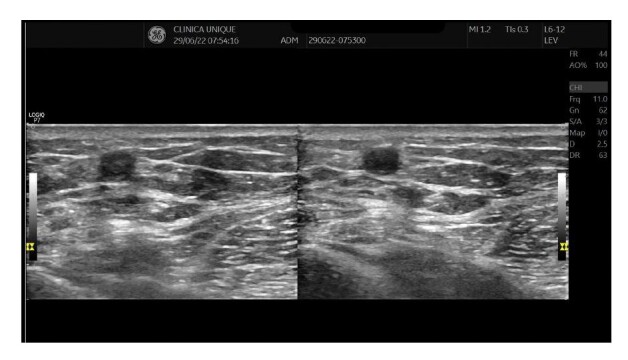
Images from the final control ultrasound scan performed 3 months after the procedure. (A) Right great saphenous vein with hyperechogenic material inside; (B) Transducer compression maneuver showing that the great saphenous vein is uncompressible.

One week after the procedure, the diameter of the ulcer had receded, with initial epithelialization at the margins, regression of the edema, and good control of the pain. The wound had fully healed by 9 weeks, with complete epithelialization of the raw area, total regression of pain and edema, and partial lightening of the ochrodermatitis, with improved quality of the skin of the distal third of the leg and the sclerotic dermatofibroma (Figure 4). The patient reported improvement of his insomnia and elimination of limitations affecting daily and occupational activities. No phlebitis or pain was observed along the path of the treated veins. No DVT was identified during ultrasonographic follow-up at 30 days, 4 months, or 1 year after the procedure. Control ultrasound revealed an uncompressible saphenous vein with no flow on duplex ultrasound, as shown in Figure 5. The treatment with Daflon® was maintained for 4 months, combined with elastic compression and skin moisturization.

## DISCUSSION

The ideal technique for treatment of varicose veins is one that achieves rapid exclusion, in an outpatients or office setting, rapid recovery, and a high success rate. A 2017 systematic review^6^ of prospective studies included 1,521 saphenous veins (1,267 great and 254 small) treated using MOCA. Anatomic success was defined as complete closure of the treated vein on follow-up duplex ultrasound and was achieved in 92% of cases 6 months after treatment, 91% at 12 and 24 months, and 87% after 3 years, showing that this is a safe and effective method for treating trunk vein incompetence.^6^ Other outcomes assessed included major complications such as DVT, pulmonary embolism, or paresthesia, which were rare (at less than 0.2% of the patients). Similarly, in the case described herein, there were no complications, and technical and anatomic success were maintained up to the most recent follow-up examination, conducted 1 year after treatment.

With regard to comparison with thermoablation methods, in 2021 a randomized clinical trial assessed 125 patients who had undergone treatment of the great saphenous vein using thermoablation (endolaser or radio frequency) or MOCA after ultrasonographic follow-up for up to 3 years.^7^ The study found that the efficacy of treatment with MOCA was inferior to the thermoablative methods (82% and 100% respectively, with p < 0.005), observing a higher rate of recanalization of saphenofemoral junctions with diameters exceeding 7 mm, similar to what is observed after foam sclerotherapy alone, after which recanalization rates increase in proportion to the diameter of the treated vein.^4,8^ The Brazilian Chronic Venous Disease Guidelines recommend using MOCA for treatment of saphenous veins (great and small), with evidence level B and recommendation class IIb; but describes inferior occlusion rates when compared to ablative techniques at 12 to 36 months of follow-up.^5^

Despite the higher rate of recanalization with MOCA, the technique does offer undeniable advantages. Some studies have reported that MOCA for the saphenous vein is less painful compared to thermoablative methods and enables earlier return to habitual activities.^9-11^ Since the ablation mechanism does not employ heat, there is no dissipation of energy to the adjacent structures, which means tumescent anesthesia is unnecessary. Moreover, the procedure has a smaller learning curve, less intraoperative and postoperative pain, and lower risk of nerve or skin damage.^12,13^ As such, mechanochemical methods can be considered a good option for treatment of the great saphenous vein below the knee or the small saphenous vein.^12^ The decision to treat the case described using MOCA was because it was necessary to treat the entire length of the great saphenous vein, including the distal third of the leg and venous ulcer bed, which was in an area where thermal ablation would increase the risk of nerve injury. The MOCA technique was also chosen because it is a painless procedure and is rapid (7 minutes in the case in question), is ideal for outpatients and office treatment, with no need for sedation or anesthesia, and at a lower total cost because it does not require hospital admission and enables a rapid return to work.

MOCA is also an option for treating patients with active venous ulcers and great saphenous vein incompetence, as in the case described. A study that compared the rate of venous ulcer healing among patients who underwent thermal ablation or MOCA of the saphenous vein assessed 82 patients (53 treated with MOCA and 29 with thermoablative methods), observing ulcer healing rates of 74% and 35%, respectively. The mean follow-up time was 12.8 months in the thermal ablation group and 7.9 months in the group treated with MOCA.^13^

Finally, a meta-analysis of randomized and prospective studies published by Smith^14^ analyzed 414 patients with venous ulcers, comparing the use of micronized diosmin and hesperidin (Daflon®), at a dosage of 1,000 mg per day, combined with conventional treatment (compression therapy and local wound care) vs. conventional treatment alone. The study demonstrated that the likelihood of healing was 32% higher with combined treatment and that the wound healed more quickly (16 weeks vs. 21 weeks).^14^ In the case in question, the venotonic was used throughout the treatment, in combination with mechanochemical treatment of venous insufficiency, and the venous ulcer healed in 9 weeks.

This case report demonstrates that venous ulcers associated with saphenous vein incompetence can be successfully treated using the mechanochemical ablation technique in an outpatients setting, rapidly, painlessly, and with immediate return to daily activities.

## Data Availability

Dados não informados ou não utilizados: “Compartilhamento de dados não se aplica a este artigo, pois nenhum dado foi gerado ou analisado.”
