# Supplementary material for: Efficacy of using the mechanochemical endovenous ablation to treat chronic venous ulcers: case report and literature review
Source: J Vasc Bras. 2025 Nov 10;24:e20230173. doi: 10.1590/1677-5449.202301732 (PMC12642920; doi:10.1590/1677-5449.202301732)
Supplement: Translation

# Translation: Eficácia da utilização da técnica de ablação mecanoquímica no tratamento e cicatrização rápida de úlcera venosa crônica: relato de caso e revisão de literatura

**DOI:** 10.1590/1677-5449.202301731

**Authors:** Arthur Souza Magnani, Thiago Faiad Name Villari, Alexandre de Azevedo Melo, Marcela Juliano da Silva, Dafne Braga Diamante Leiderman

**Affiliations:** 1 Hospital Israelita Albert Einstein HIAE, São Paulo, SP, Brasil.; 2 Hospital Estadual Ipiranga, São Paulo, SP, Brasil.; 3 Universidade de São Paulo USP, Faculdade de Medicina, São Paulo, SP, Brasil.; 4 Instituto de Assistência Médica ao Servidor Público do Estado de São Paulo IAMSPE, São Paulo, SP, Brasil.

**Keywords:** úlcera venosa, ablaçāo mecanoquímica, Flebogrif®, espuma de polidocanol

## Abstract

Este relato de caso apresenta o tratamento bem-sucedido de um paciente do sexo masculino, de 47 anos, com úlcera venosa de membro inferior associada à insuficiência venosa profunda e superficial. O paciente apresentava úlcera venosa crônica, dermatite ocre e dor, o que prejudicava suas atividades laborais. O ultrassom Doppler revelou insuficiência venosa profunda, incompetência da veia safena magna e perfurantes indiretas no leito da úlcera. Foi realizada ablação mecanoquímica da veia safena magna com Flebogrif®. A úlcera venosa cicatrizou em 9 semanas após o procedimento e mantém-se cicatrizada no acompanhamento de 1 ano. Houve melhora dos sintomas, sem complicações relacionadas ao procedimento. Este relato de caso destaca a eficácia da ablação mecanoquímica no tratamento de úlceras venosas, mesmo na presença de insuficiência venosa profunda.

## INTRODUÇÃO

A prevalência estimada da doença venosa crônica varia de acordo com a região geográfica, sendo mais elevada em países ocidentais, onde pode atingir até 40% da população adulta feminina e 17% da masculina^1^. No Brasil, cerca de 62% das mulheres e 37% dos homens acima de 30 anos são acometidos por essa doença^2^. Entre os fatores de risco estabelecidos destacam-se sexo feminino, idade, gravidez, histórico familiar de doença venosa crônica, obesidade e ocupações que exijam ortostatismo prolongado^3^.

As novas técnicas surgiram para o tratamento da insuficiência da veia safena magna, com o objetivo de melhorar a recuperação pós-operatória observada na fleboextração convencional. A termoablação por radiofrequência ou endolaser é atualmente considerada o padrão-ouro, com estudos demonstrando sucesso técnico em 90 a 100% dos casos^4^, corroborando a indicação da Diretriz Brasileira de Doença Venosa Crônica da Sociedade Brasileira de Angiologia e de Cirurgia Vascular^5^. A escleroterapia com espuma de polidocanol provoca lesão química na parede do vaso, porém apresenta uma taxa de recanalização mais elevada (de 51,2% a 74,2% em 3 anos) em veias safenas com diâmetro superior a 6 mm^4^.

A ablação mecanoquímica (*mechanochemical ablation*, MOCA) surgiu com o objetivo de ser um tratamento indolor, por ser um método não térmico e não tumescente, aumentando a taxa de sucesso técnico observada no tratamento com escleroterapia isolada. A MOCA combina a lesão química causada pela espuma com a lesão mecânica induzida por tração com ganchos radiais retráteis, como no caso do Flebogrif® (Balton, Varsóvia, Polônia) ou por rotação de fios giratórios, como no Clarivein® (Merit Medical Systems Inc., South Jordan, EUA)^4^.

Neste artigo, relatamos o caso de um paciente com úlcera venosa em membro inferior, tratado com sucesso com Flebogrif®. O relato deste caso foi avaliado e aprovado pela comissão de ética competente (CAAE 80173524.0.0000.0071, parecer consubstanciado número 6.859.125) e seguiu as diretrizes CAse REport (CARE).

## RELATO DE CASO

Paciente do sexo masculino, 47 anos, negro, tabagista e hipertenso em uso de anlodipino e losartana, com queixa de úlcera na perna direita há 2 meses, sem antecedentes pessoais de trombose venosa profunda (TVP). A úlcera havia surgido de forma espontânea, com aumento progressivo, associado a edema, hipercromia do membro e dor intensa, que causava insônia e prejudicava as atividades laborais que exigiam longos períodos de ortostase.

Foi realizado tratamento clínico com antibioticoterapia em ciclos de cefazolina, amoxicilina-clavulanato e clindamicina associadas a ciprofloxacino, além do uso de pomadas tópicas como Diprogenta® (Anápolis, Brasil) e colagenase, por orientação de clínicos gerais, porém sem melhora do quadro.

No exame físico, observaram-se veias varicosas no terço distal da perna direita, associadas a dermatite ocre, edema e úlcera na face medial do tornozelo, com aproximadamente 10 cm em seu maior diâmetro. A úlcera não tinha aspecto isquêmico ou infeccioso, apresentando bom tecido de granulação (Figura 1). Todos os pulsos (femorais, poplíteos e tibiais) eram palpáveis bilateralmente.

**Figura 1:** Úlcera venosa em face medial do tornozelo. O steri-strip indica o ponto de punção da veia safena magna para tratamento com Flebogrif®.

Foi realizado ultrassom Doppler venoso dos membros inferiores em ortostase, que evidenciou insuficiência dos sistemas venosos profundo (refluxo superior a 1 segundo na veia poplítea) e superficial, com insuficiência da junção safeno-femoral e ao longo de todo o trajeto da veia safena magna durante as manobras de Valsalva e compressão distal (Figura 2). Os diâmetros da veia safena magna da coxa proximal e distal eram de 0,80 e 0,67 cm respectivamente. Já o diâmetro da veia safena magna da perna era de 0,50 cm, tanto no segmento mais proximal quanto no tornozelo. Também foram observadas veias tributárias e perfurantes indiretas insuficientes na topografia da úlcera.

**Figura 2:** Imagem do ultrassom Doppler na consulta inicial demonstrando refluxo significativo da veia safena magna.

Primeiramente, foi otimizada a analgesia com dipirona, naproxeno e pregabalina, associado a curativo com loção oleaginosa por 3 dias, e introduziu-se diosmina + hesperidina micronizada (Daflon®, Laboratórios Servier do Brasil, Rio de Janeiro, Brasil) na dose de 1.000 mg por dia. Optou-se pelo tratamento ambulatorial da veia safena magna direita com Flebogrif®. Foi realizado botão anestésico com lidocaína a 2% sem vasoconstritor, somente no local da punção ecoguiada da veia safena magna direita, ao nível do maléolo medial. Em seguida, realizou-se nova punção no mesmo local anestesiado e, com o auxílio do fio-guia e do ultrassom, o cateter Flebogrif® foi posicionado na junção safeno-femoral, com liberação dos ganchos retráteis (Figura 3).

**Figura 3:** Imagem de ultrassom intraoperatório mostrando o cateter Flebogrif® com as garras abertas ao nível da junção safeno-femoral.

Foi empregada a técnica padronizada com espuma de polidocanol a 3%, conforme a técnica de Tessari (1 mL de polidocanol a 3% misturado com 4 mL de ar). A espuma foi injetada à medida que o cateter Flebogrif® era tracionado. Cerca de 1 mL de espuma era administrado a cada 5 cm de extensão de veia percorrida pelo cateter, totalizando 13 mL de espuma, sem intercorrências. Foi realizada punção direta ecoguiada de tributária varicosa ao nível da ferida, com injeção de 2 mL de espuma de polidocanol a 1%, conforme técnica de Tessari (1 mL de polidocanol a 1% misturado com 4 mL de ar). O procedimento teve duração total de 7 minutos, e o controle ultrassonográfico final identificou que espuma se distribuiu de forma homogênea em todo o trajeto da veia safena magna, assim como nas tributárias e perfurantes no local.

Ao final do procedimento, foi realizado curativo com HydroClean® (Hartmann Brasil, São Gonçalo, Brasil) e filme transparente associado a meia elástica rígida de alta compressão (30-40 mmHg), comprimento 7/8. Após 3 dias, esse curativo foi substituído por malha de gaze vaselinada, mantendo-se o uso diário da meia elástica.

Uma semana após o procedimento, houve redução do diâmetro da úlcera, com epitelização inicial das bordas, regressão do edema e bom controle da dor. A cicatrização completa da ferida aconteceu em 9 semanas, com epitelização completa da área cruenta, regressão total da dor e do edema e clareamento parcial da dermatite ocre, com melhora da qualidade da pele do terço distal da perna e da dermatofibroesclerose (Figura 4). O paciente relatou melhora da insônia e ausência de limitações para as atividades diárias e laborais. Não foram observadas flebite ou dor no trajeto das veias tratadas. Tampouco foi identificada TVP durante o acompanhamento ultrassonográfico aos 30 dias, 4 meses e 1 ano após o procedimento. Realizou-se ultrassom de controle, que evidenciou veia safena incompressível e sem fluxo ao Doppler, conforme registrado na Figura 5. Manteve-se o tratamento com Daflon® por 4 meses, associado a elastocompressão e hidratação da pele.

**Figura 4:** Úlcera cicatrizada 9 semanas após o procedimento de ablação mecanoquímica.

**Figura 5:** Imagem final do exame de ultrassom de controle realizado 3 meses após o procedimento. (A) Veia safena magna direita com material hiperecogênico em seu interior; (B) Manobra de compressão com o transdutor evidenciando veia safena magna incompressível.

## DISCUSSÃO

A técnica ideal para o tratamento de varizes é aquela que permite excussão rápida, abordagem ambulatorial, recuperação rápida e alta taxa de sucesso. Uma revisão sistemática^6^ com estudos prospectivos de 2017 incluiu 1.521 veias safenas (1.267 magnas e 254 parvas) tratadas com MOCA. O sucesso anatômico, definido como a obliteração completa da veia tratada ao ultrassom Doppler de controle, foi obtido em 92% dos casos após 6 meses do tratamento, 91% após 12 e 24 meses e 87% após 3 anos, demostrando que se trata de um método eficaz e seguro para o tratamento da insuficiência troncular^6^. Outros desfechos avaliados incluíram complicações maiores como TVP, embolia pulmonar ou parestesia, que foram raras (menos de 0,2% dos pacientes). Assim como no caso aqui descrito, não houve complicações, e o sucesso técnico e anatômico obtido manteve-se até o último exame, realizado 1 ano após o tratamento.

Em comparação aos métodos termoablativos, um ensaio clínico randomizado de 2021 avaliou 125 pacientes submetidos ao tratamento da veia safena magna com termoablaçāo (endolaser ou radiofrequência) e com MOCA após acompanhamento ultrassonográfico por até 3 anos^7^. A eficácia do tratamento com MOCA nesse estudo foi inferior à dos métodos termoablativos (82% e 100% respectivamente, com p < 0,005), observando-se maior taxa de recanalização em junções safeno-femorais com diâmetro superior a 7 mm de diâmetro, semelhante ao que ocorre na escleroterapia com espuma isolada, em que as taxas de recanalização aumentam proporcionalmente ao diâmetro da veia tratada^4,8^. A Diretriz Brasileira de Doença Venosa Crônica recomenda o uso da MOCA para o tratamento das veias safenas (magna e parva), com nível de evidência B e classe IIb; porém, relata taxas de oclusões inferiores quando comparado às das técnicas ablativas, em seguimentos de 12 a 36 meses^5^.

Apesar da maior taxa de recanalização com MOCA, a técnica apresenta vantagens indiscutíveis. Alguns estudos apontam que a MOCA da veia safena é menos dolorosa quando comparada aos métodos termoablativos e permite retorno precoce às atividades habituais^9-11^. Por não utilizar o calor como mecanismo de ablação, não há dissipação de energia para as estruturas adjacentes, o que dispensa o uso de anestesia tumescente. Ademais, o procedimento tem curva de aprendizado menor, menos dor intra e pós-operatória e menor risco de lesão nervosa ou cutânea^12,13^. Por isso, os métodos mecânico-químicos podem ser considerados uma boa alternativa para o tratamento da veia safena magna infragenicular ou da veia safena parva^12^. A opção pelo tratamento com MOCA no caso descrito deveu-se à necessidade de tratar a veia safena magna em toda a sua extensão, incluindo o terço distal da perna e o leito da úlcera venosa, região em que a termoablação aumentaria o risco de lesão nervosa. A técnica MOCA também foi escolha por ser um procedimento indolor e rápido (7 minutos no caso em questão), ideal para tratamento ambulatorial, sem necessidade de sedação ou anestesia, com menor custo total por dispensar internação hospitalar e possibilitar rápido retorno ao trabalho.

A MOCA também é uma opção de tratamento para pacientes com úlcera venosa ativa e insuficiência da veia safena magna, como no caso relatado. Um estudo que comparou a taxa de cicatrização de úlcera venosa em pacientes submetidos a ablação térmica ou MOCA da veia safena avaliou 82 pacientes (53 tratados com MOCA e 29 com métodos termoablativos), evidenciando taxas de cicatrização de 74% e 35%, respectivamente. O tempo médio de acompanhamento foi de 12,8 meses no grupo da termoablação e de 7,9 meses no grupo tratado com MOCA^13^.

Por fim, a metanálise de estudos randomizados e prospectivos publicada por Smith^14^ analisou 414 pacientes com úlcera venosa, comparando o uso combinado de diosmina-hisperidina micronizada (Daflon®), na dose de 1.000 mg por dia, associado ao tratamento convencional (terapia compressiva e cuidados locais com a ferida) *versus* o tratamento convencional isolado. O estudo demonstrou que a chance de cicatrização da úlcera foi 32% maior com a terapia combinada, além de uma cicatrização mais rápida da ferida (16 semanas *versus* 21 semanas)^14^. No caso em questão, foi usado venotônico durante todo o tratamento, associado ao tratamento mecanoquímico da insuficiência venosa, com cicatrização da úlcera venosa em 9 semanas.

O presenta relato evidenciou que úlceras venosas associadas a insuficiência da veia safena podem ser tratadas com sucesso pela técnica ablativa mecanoquímica de forma ambulatorial, rápida e indolor, com retorno imediato às atividades diárias.

## Data Availability

Data not reported or used: “Data sharing does not apply to this article, as no data were generated or analyzed.”
